# Building integrated care systems: a case study of Bidasoa Integrated Health Organisation

**DOI:** 10.5334/ijic.1796

**Published:** 2015-06-24

**Authors:** Nuria Toro Polanco, Iñaki Berraondo Zabalegui, Itziar Pérez Irazusta, Roberto Nuño Solinís, Mario Del Río Cámara

**Affiliations:** O+Berri, Basque Institute for Healthcare Innovation, Basque Country, Spain; Quality Assurance and Procurement, Ministry of Health, Basque Country, Spain; Bidasoa Integrated Health Organisation, Osakidetza, Basque Country, Spain; Deusto Business School, University of Deusto, Basque Country, Spain; O+Berri, Basque Institute for Healthcare Innovation, Basque Country, Spain

**Keywords:** integrated care, Chronic Care Model, Basque Country, health organisation, Triple Aim

## Abstract

**Introduction:**

This paper analyses the implementation of integrated care policies in the Basque Country through the deployment of an Integrated Health Organisation in Bidasoa area during the period 2011–2014. Structural, functional and clinical integration policies have been employed with the aim to deliver integrated and person-centred care for patients, especially for those living with chronic conditions.

**Methods:**

This organisational case study used multiple data sources and methods in a pragmatic and reflexive manner to build a picture of the organisational development over a 4-year period. In order to measure the progress of integration three concepts have been measured: (i) readiness for chronicity measured with Assessment of Readiness for Chronicity in Healthcare Organisations tool; (ii) collaboration between clinicians from different care levels measured with the D'Amour Questionnaire, and (iii) overall impact of integration through several indicators based on the Triple Aim Framework.

**Results:**

The measurement of organisational readiness for chronicity showed improvements in five of the six areas under evaluation. Similarly the collaboration between professionals of different care levels showed a steady improvement in each of the 10 items. Furthermore, the Triple Aim-based indicators showed a better experience of care in terms of patients’ perceptions of care coordination; a reduction in hospital utilisation, particularly for patients with complex chronic conditions; and cost-containment in terms of per capita expenditure.

**Conclusion:**

There is a significant amount of data that shows that Bidasoa Integrated Health Organisation has progressed in terms of delivering integrated care for chronic conditions with a positive impact on several Triple Aim outcomes.

## Introduction

In 2010 when the “Strategy to tackle the challenge of chronicity in the Basque Country” [1] was launched, it was the first time in which an integrated care strategy was going to be promoted on a formal basis in the Basque Health System. Even though an explicit definition of integrated care was not included in the Strategy, several theoretical frameworks were taken into account to guide transformational leaders towards the implementation of integrated care. Of particular importance were the Chronic Care Model [2], the work on Integrated Delivery Systems [3] and approaches emphasising the need of bottom-up approaches for effective clinical integration [4]. The emerging narrative of transformation highlighted the need of integrated and person-centred care for patients living with chronic conditions [5]. Within this strategic framework, two types of integrated care implementation approaches were put in place in the Basque Health System.

On the one hand, bottom-up implementation focused on clinical and functional integration [6, 7], which looks for coordination of care processes between primary and secondary care, particularly through new models of care for complex chronic patients and through the design and implementation of clinical pathways for high impact and prevalent chronic conditions such as diabetes, chronic obstructive pulmonary disease (COPD), heart failure, etc. The introduction of nurse case managers and liaison nurses [8, 9] facilitated the coordination and transitional tasks needed for these innovative approaches. Several processes and tools were established to promote local innovation, support clinical leaders, create alignment, build capabilities, evaluate the results and progress of these interventions and facilitate the spread of innovation.

**Figure 1.**
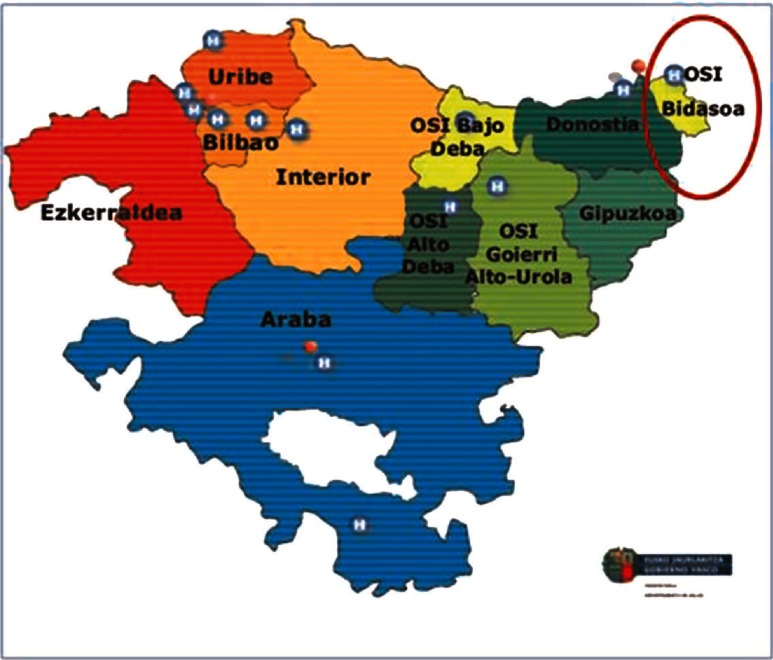
Location of Bidasoa IHO (OSI Bidasoa) in the Basque Country

On the other hand, organisational integration was also promoted with the aim of merging hospital and primary care structures under one single organisation [10]. This approach resulted in the creation of several Integrated Healthcare Organisations (IHOs) starting in 2011. In order to draw a map of the potential Integrated Health Organisations in which the Basque Health structure could be divided, hospitals were matched with the primary care organisations within their geographical area of influence. It was also agreed to conduct a pilot study and create the first Integrated Health Organisation between Bidasoa Hospital and the health centres that depend on it in January 2011, which is the focus of this case study (Figure 1).

This strategy was considered successful and in 2013, the “Integrated Care Plan for the Basque Country” was released aiming at transforming all the health organisations that belong to Osakidetza/Basque Health Service into IHOs, 13 in total, so that by the end of 2016 primary and secondary care centres will be working under the same organisational and managerial structure.

The vision behind the creation of Integrated Health Organisations was to achieve more efficiency and better quality of care for their catchment populations within a Triple Aim logic [11]. The converging narratives of integrated care and better chronic care provide the underlying evidence to this endeavour [12, 13]. To evaluate its progress, the Basque Institute for Healthcare Innovation and Osakidetza developed and applied a number of assessment tools in order to: (i) monitor organisational advances in achieving several process indicators and intermediate outcomes [14], (ii) assess how developed organisational and clinical systems are towards the provision of integrated care for chronic conditions [15], (iii) know the degree of collaboration between clinical staff from primary and secondary care [16].

However, these instruments were not concurrently used and their results compared, nor have their results been linked to patient-, provider- and system-level outcomes. This paper is an attempt to link those assessment tools and try to put them in relation with key outcomes, to see whether or not structures, processes and results are aligned as a consequence of structural, functional and clinical integration.

## Development of integrated care in the Basque Country

The Basque Health System is a typical Beveridge model, characterised by universal access for all citizens. As is the case of other Spanish regions, the Basque Country holds health planning powers as well as the capacity to organise and manage its own health services. There are two main bodies: on the one hand, the Basque Ministry of Health, that is responsible for funding, planning, managing and regulating the health system, and on the other hand “Osakidetza”, the Basque Health Service, is the organisation in charge of providing public health care services.

Traditionally, primary care organisations and hospitals have been run on a separate basis. In fact, primary care, which acts as the gatekeeper of the system, was managed until 2011 through Primary Care Organisations (seven across the Basque Country) that comprised all primary care centres within a specific geographical area with a population ranging from 200,000 to 400,000 citizens. The services provided by primary care organisations and hospitals were commissioned through a tool called the Contract-Program in an independent manner (no shared objectives between different levels of care).

In 2015, 11 Integrated Health Organisations are operating and this integration process will end in 2016 with 13 IHOs delivering integrated health services to the whole population of the Basque Country with catchment areas ranging from 30,000 to 400,000 citizens.

It should be stressed that integration operates at different levels. At the strategic level, IHOs are ruled by an integrated strategic plan, which sets common goals for both primary and secondary care. These plans were developed at the Integrated Health Organisation level with the involvement and participation of staff representatives.

The integration process also includes the funding mechanisms. There is a single source of funding for both primary and secondary care and it is negotiated between each of the Integrated Health Organisations and the Health Ministry. Even though adjusted capitation mechanisms are not yet in place for the all Integrated Health Organisations, a number of pilots have been launched in order to test its applicability to the whole system.

Following Kaiser Permanente pyramid and NHS risk-stratification initiatives [17, 18], the whole population has been stratified through models predicting future (next year) health care costs [19]. To increase functional coordination, integrated clinical pathways have been designed and implemented between primary and secondary care professionals for chronic conditions such as diabetes, COPD and heart failure among others. In order to better manage patients with complex health needs, some organisations have developed Continuity of Care Units, in which primary care doctors, internal medicine specialists, case managers and social workers work in multidisciplinary teams to provide higher quality care to complex chronic patients [20, 21]. In relation to information systems, a shared electronic medical record has been fully developed, so that patient information can be exchanged between professionals from different care levels.

## Bidasoa Integrated Health Organisation

Bidasoa Integrated Health Organisation was the first integrated health care organisation, created in January 2011. It was considered a landmark for the Basque Health Service in terms of building integrated care. It comprised the Bidasoa Hospital which has 96 beds, and 3 primary care centres (Irun, Hondarribia and Dumboa), next to the French border. The IHO catchment population is around 90,000 citizens.

As it happened with other organisations, care delivery in Bidasoa area was fragmented, with poor communication between primary and secondary care professionals. The main objective for the new organisation was to improve the quality of care through evidence-based practices and the enhancement of the coordination between levels, trying to be as efficient as possible and taking into account that patients must be the centre of the whole process. Managers in Bidasoa defined three axes on which to build the new organisation model: culture, clinical practice and governance. They used the following definition for care integration “the process that involves creating and maintaining, over time, a common structure between independent stakeholders (and organisations) for the purpose of coordinating their interdependence in order to enable them to work together on a collective project” [22].

With regard to culture, a more patient-oriented approach based on collaborative practices was sought, through deeper mutual knowledge and enhanced communication between professionals of both levels of care. In order to improve clinical practice, technical boards were created, as well as mixed clinical committees, so that issues such as patient safety and palliative care among others were approached and managed in multidisciplinary teams. The first Continuity of Care Unit to treat complex chronic patients was created in Bidasoa Integrated Health Organisation, in which more than 200 patients are being taken care nowadays. Briefly the Continuity Care Unit (CCU) worked as follows: the referral internist (one for each health centre) is responsible for the admission of patients with complex or multiple conditions in the event that they require admission to hospital. The mission of the CCU is to stabilise patients and facilitate continuity of care by the general practitioner. These patients have in place a continuity of care plan between levels of care and they are admitted to the hospital through a special circuit (not A&E) when needed. The role of the liaison nurse is to support the patient in his/her transition from hospital to home, where they are followed up by the general practitioner. The referral internist visits the health centre every other week to undertake clinical sessions with the primary care staff professionals, and is available to general practitioners at any time for any queries they may have.

As far as governance is concerned, a clinical governance unit was created in the area of paediatrics to support the joint work and processes between primary and secondary professionals of that area. Besides, a shared electronic medical record that facilitates the provision of coordinated care is now fully developed. Furthermore, new agreements will be reached to set common clinical and quality performance indicators for both levels of care. In brief, Bidasoa Integrated Health Organisation shows all the elements that feature the Basque integrated health care strategy in which collaboration is one of the cornerstones.

## Evaluation tools

**Figure 2.**
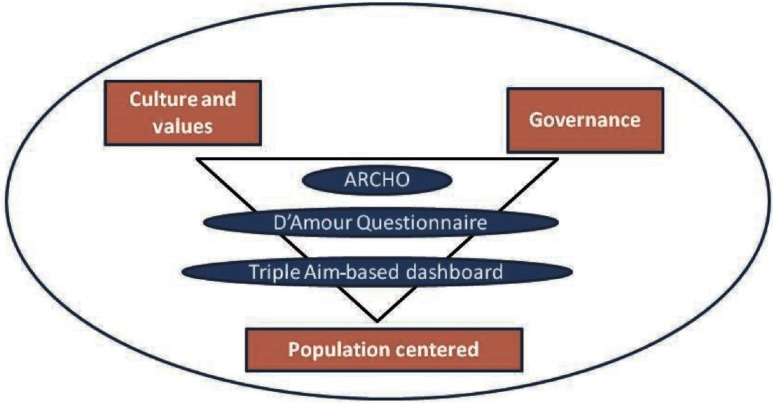
Evaluation tools within the three axes

Since its creation, managers of Bidasoa Integrated Health Organisation have been concerned about the evaluation of the performance of the organisation as well as its advances in providing integrated care. Three main assessment tools have been applied during the post-integration period (Figure 2): (i) Assessment of Readiness for Chronicity in Healthcare Organisations (ARCHO) has been designed [23], (ii) D'Amour which is a questionnaire to assess collaboration between primary and specialised care health care professionals [24] and (iii) a Triple Aim-based dashboard. These tools are explained in detail in the following paragraphs.

### Assessing integrated care using ARCHO

ARCHO enables health care organisations to self-assess the readiness to provide integrated care to cope with chronicity. It helps organisational teams evaluate changes in chronic illness care processes and guide improvement efforts through the provision of a roadmap of interventions to improve integrated care management. Moreover, it also evaluates the level and nature of improvement in the system. ARCHO is based on six areas/dimensions of system change suggested by the Chronic Care Model [25] and 80 interventions or items. In relation to its rating scale, it goes from 0 to 100 points in each dimension.

The six dimensions of ARCHO are: (i) “Organisation of the Health System”, which evaluates whether there is a strategic direction towards the provision of integrated care; (ii) “Community Health”, which represents the existence of links with community resources; (iii) “Healthcare Model”, that measures how planned, proactive and coordinated the provision model is; (iv) “Self-management”, that measures up to what extent patients are being empowered to be able to self-manage their condition; (v) “Clinical Decision Support”, which takes into account the capacity of the system to improve health outcomes using decision support tools, training of professionals and exchange of knowledge among providers. And last but not least, (vi) “Information Systems”, that measure the extent to which information and communication systems are integrated and oriented to improve patients care.

Assessing the level of Integration of your organisation using ARCHO requires minimum training and all instructions are available in English in the official website: http://www.iemac.es/cuestionario.asp. In the case of Bidasoa, it has been used to assess an organisation situated in the macro level, but it can also be used to evaluate integration for services (meso) or teams (micro). It requires a multidisciplinary team and takes around 5–6 hours to reach consensus about the 80 items of the tool.

### Collaboration between professionals of different care levels: D'Amour Model

**Figure 3.**
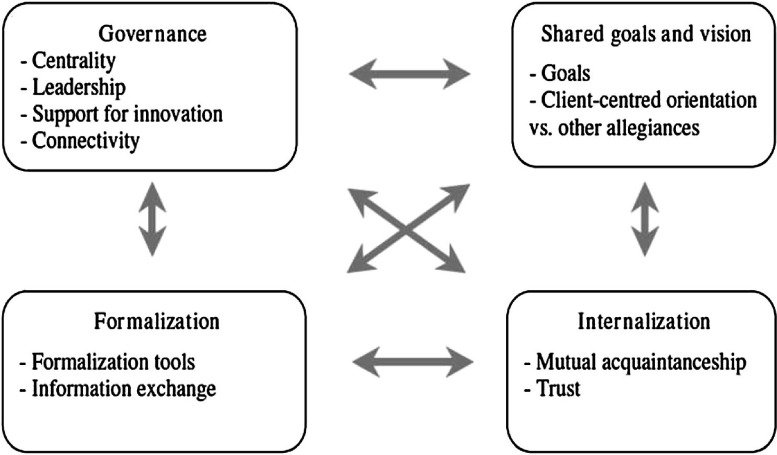
D'Amour Model of collaboration

Achieving integrated care requires not only the implementation of new structures but also the development of new clinical practices based on collaboration [26]. The D'Amour Model is based on the concept of collective action. The basic premise is that professionals want to work together to provide better care. The model suggests that it is possible to analyse collective action in four dimensions that are operationalised by 10 indicators (Figure 3). Two of the dimensions involve the relationship between professionals and two involve the organisational setting.

The relational dimensions are: “Shared Goal and Visions”, which refers to the existence of common goals and whether or not they are embraced by the team and “Internalization”, which refers to the existence of awareness among professionals of their interdependencies. The organisational dimensions are: “Formalisation”, or the extent to which documented procedures to communicate desired outputs and behaviours exist and are being used. And “Governance”, related the existence of a leadership structure that supports collaboration.

These dimensions are assessed via a questionnaire that is sent to health professionals within the same IHO and has a score that ranges from 0 to 5 and it takes around 10 minutes to complete. These four dimensions together and the interaction between them capture the idea behind the concept of collective action suggested by the D'Amour Model.

## Measuring impact: process evaluation

### ARCHO: results

**Figure 4.**
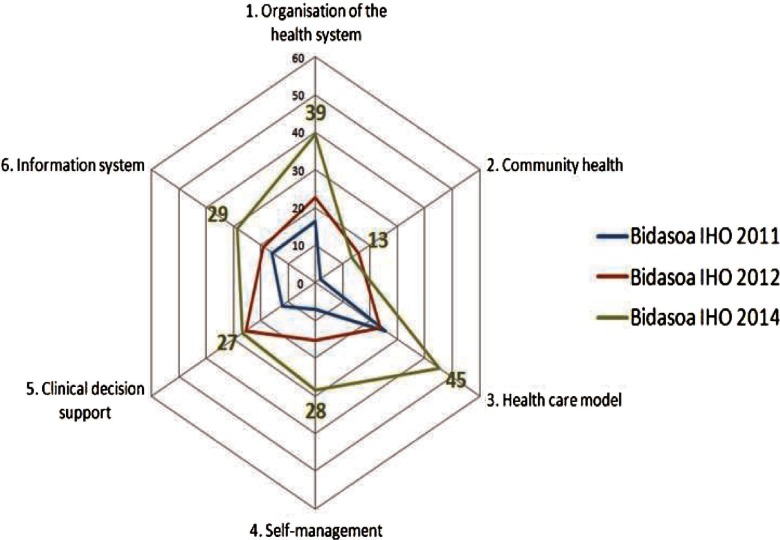
Evolution in ARCHO scores in Bidasoa Integrated Health Organisation by dimension

When it comes to analysing the score of Bidasoa Integrated Health Organisation in ARCHO, Figure 4 shows data from 2011, 2012 and 2014. As we can see in Figure 4, there has been an improvement in all dimensions, except for the dimension two, “Community Health” in which it has been a decrease since 2012, due to the loss of agreements with community agents. The organisation is doing very well in “Healthcare Model”, with a great development of clinical pathways, case managers and hospital liaison nurses, and the relevant role of the Continuity of Care Unit to treat complex patients. As it is reflected in the Dimension 1, the macro level shows an increasing support towards integrated care. It is interesting to emphasise how self-management support has been growing during the last years.

However it is interesting to note that there has been a decrease in Dimension 2 “Community Health”. The feedback we have had from Bidasoa Executive Board is that one of the items in this dimension which relates with the coordination between health and social care, has not been properly addressed until the end of 2014, when a Committee for Health and Social Care Coordination was created.

Lastly, it should be highlighted that improvements in ARCHO scores are not related to the structural changes such as the creation of the IHO (at most this fact can be deemed as a facilitator). For instance, the improvements in Dimension 6 (information system) are related with the implementation of the “Unified Clinical Record” for both primary and secondary care.

### D'Amour Model: results

**Figure 5.**
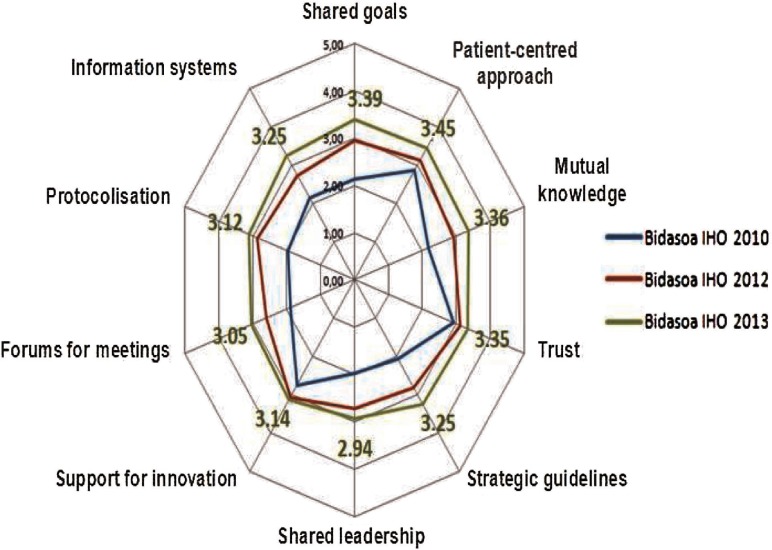
Results in collaboration between professionals of different care levels in Bidasoa Integrated Health Organisation

Stemming from D'Amour Model, Osakidetza and the Basque Institute for Healthcare Innovation elaborated a questionnaire [24], to measure the degree of collaboration between clinical staff of different care levels. Bidasoa Integrated Health Organisation has applied this questionnaire three times (in 2010 before integration, 2012 and 2013) using a sample (number of clinicians) of 80 in 2010, 117 in 2012 and 122 in 2013. The results are shown in Figure 5.

There has been a positive evolution in all items according to the spider graph (Figure 5). Almost all of them show scores higher than 3 (except for shared leadership). Patient-centred approach is at the top.

The improvement in Figure 5 between 2010 and 2013 has been the result of a new culture of collaborative working between primary and secondary care. Clinical sessions for professionals from both levels of care have been promoted, a Care Continuity Unit for Chronic Patients in which both levels take part has been created and the Training Unit has started to offer courses where both levels of care participate.

**Figure 6.**
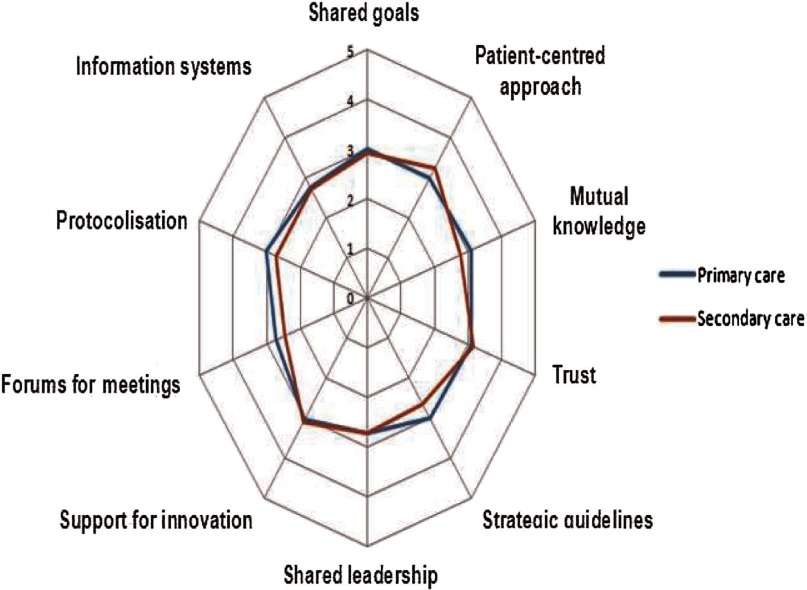
Results in collaboration between professionals of different care levels in Bidasoa Integrated Health Organisation: primary and secondary care professionals, 2012

Since there are available data for primary and secondary care professionals, it is interesting to analyse whether there are differences in the perceptions of both groups. For the year 2012, as we can see in Figure 6, there is just a slight difference between them, which suggests that the perception of the integration process is similar among both levels of care. It was conducted in 2012 to allow us to see the effects of integration.

## Measuring impact: outcome evaluation

When referring to outcomes, there is a need to take into account the simultaneous pursuit of three aims: health status of a defined population, experience of care and per capita costs of health care [11]. It is the IHO, the integrator, the one that accepts the challenge to fulfil the three aims and improve integrated care. Since there is not a single definition of the core metrics to be used for each dimension of the Triple Aim, we have selected indicators linked to the Health Plan of the Basque Health Department [7] and to existing performance measures in Osakidetza.

### Health status of the population

Life expectancy and mortality rates are commonly chosen as indicators to measure health status. Therefore we have chosen not to include these as indicators since they cannot be attributed to the implementation of the Integrated Health Organisation. In order to measure the health status of the population, a longer study and a control group should be established. It would be desirable for future research that health indicators linked to ARCHO were used in order to better correlate integration with health outcomes.

### Experience of care

For measuring the experience of care, two perspectives were considered: first, the perspective of the patient/client in his/her interaction with the health care system (“patient satisfaction and patient experience surveys”), and second, the quality of the services the health care system provides in order to design a well-coordinated experience for patients, that takes into account “hospital use and effectiveness”, “coordination between primary care and secondary care” and the care received by “patients with complex or multiple conditions” (see Table 1 for an overview of the most relevant indicators).

#### Patient satisfaction survey

Since 2011, Bidasoa Integrated Health Organisation uses a patient satisfaction survey on an annual basis in which several aspects of care are assessed by patients. In 2013, 295 patients participated in the survey. Coordination between levels of care was one of the items assessed among others. Regarding this, 87% percent of patients stated that Hospital and Primary care coordination was good/very good and 66% that coordination between Health and Social services was good/very good.

#### Hospital utilisation

**Figure 7.**
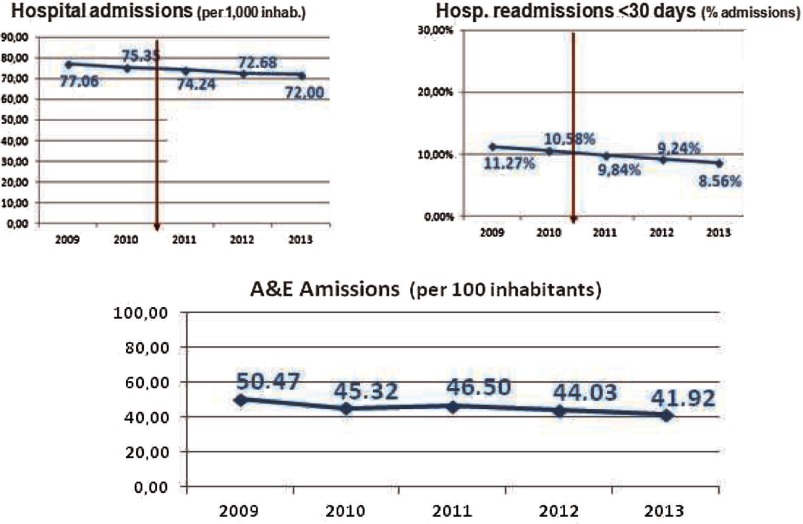
Hospital utilisation in Bidasoa Integrated Health Organisation

As the US Institute of Medicine points out in its “Crossing the Quality Chasm: A New Health System for the 21st Century” report [27] health care should match science, with neither overuse nor underuse of the best available techniques, which includes medications, procedures, surgeries, technologies and visits. Three indicators, hospital admissions, hospital readmissions within 30 days and accident and emergency admissions have been selected to describe hospital use and effectiveness in the Bidasoa Integrated Health Organisation (Figure 7).

Regarding hospital admissions, there has been a decreasing trend since 2009, ranging from 77 admissions per 1000 inhabitants to 72 in 2013 (−7%). Similarly, hospital readmissions show a decreasing trend (−24%) that place the organisation in a good position in relation to other European countries, such as England, for which readmissions for patients older than 16 was about 10% [28]. In a similar way, A&E admissions have dropped from a peak of 50 in 2009 to 42 in 2013 (−16%), which means they are performing better than Spain in this area [29].

#### Coordination between primary and secondary care

**Figure 8.**
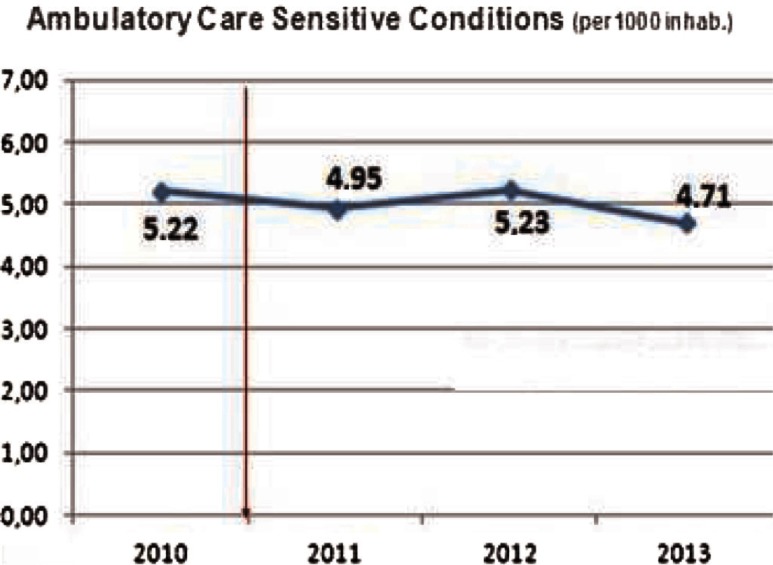
Ambulatory Care Sensitive Conditions in Bidasoa Integrated Health Organisation

Ambulatory Care Sensitive Conditions (ACSCs) are conditions for which effective management and treatment should prevent admission to hospital [30], high levels of admissions for ACSCs often indicate poor co-ordination between the different elements of the health care system, in particular between primary and secondary care. Emergency admissions for an ACSC are a sign of the poor overall quality of care, even if the ACSC episode itself is managed well. Looking at Figure 8, we see how ACSCs in Bidasoa Integrated Health Organisation have moderately fallen since 2010, standing at 4.7 in 2013 (−10%), which means a decreasing trend that favours better coordination between primary and secondary care professionals. Comparing these data with other relevant sources [30], countries such as England we get to the conclusion that the organisation is performing outstandingly in this area.

#### Patients with complex or multiple conditions

One of the most promising figures that we have seen during the assessment of the Bidasoa Integrated Health Organisation is the results on patients with multiple or complex conditions. The data used in this section come from the internal IT system of Bidasoa Integrated Health Organisation; it has not been published and has only been used internally. However it has been considered relevant for this paper and therefore has been included in this section. The study is in itself a longitudinal study pre- and post-integration with 211 patients. The results show a decrease in the rate of hospital admissions (−38%) and A&E admissions (−31%), compared to a slight decrease that we have seen previously for the whole population. This tends to confirm that those who benefit more from integrated care are those who need it most.

## Per capita costs

**Figure 9.**
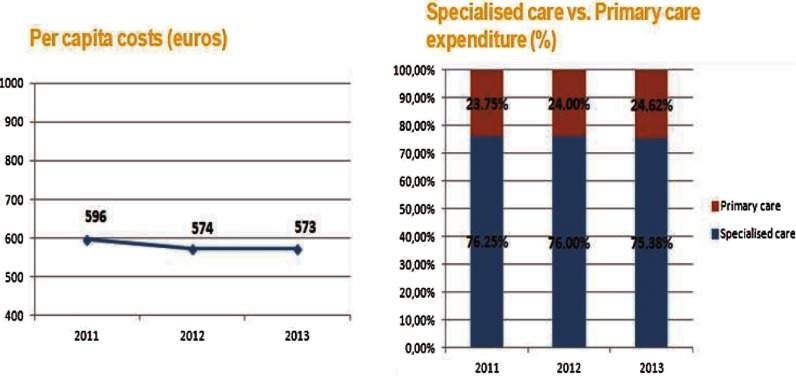
Per capita cost in Bidasoa Integrated Health Organisation and budget allocation

Reaching a good health status of the catchment population and providing them with a good experience of care should be complemented with an efficient approach in which per capita costs are reduced as much as possible without compromising health quality. In Bidasoa Integrated Health Organisation, there has been a decreasing trend in relation to costs since integration of both levels of care began as we can see on Figure 9. However, we could not directly attribute the drop in costs to integrated care [31] but to budget restrictions that are taking place in the Basque Country and all over Europe [32]. It should also be noticed that 573 Euros of per capita seems rather modest taking into consideration that the average per capita spending for health in Spain was 2199 Euros [33]. The reason for such a low figure is that the Hospital of Bidasoa does not provide tertiary care, which is the most expensive of all levels of care. Patients that need tertiary care are referred to the Hospital of Donostia, which belongs to another Integrated Health Organisation. Regarding budget allocation, we can appreciate modestly how primary care is gaining more power with respect to secondary care and it will be desirable to analyse if this trend continues over the following years.

## Discussion

Three years after the structural integration, Bidasoa Integrated Health Organisation has achieved a significant improvement in terms of delivering integrated care for chronic conditions measured by ARCHO, particularly in dimensions such as “Organisation of the Health System” and “Healthcare Model”. It is interesting to emphasise the relevance of these two dimensions. On the one hand, “Organisation of the Health System” is related to the adoption of the vision promoted in the Basque Chronicity Strategy. It considers integrated care as a priority and is acting accordingly in terms of political support and strategic planning [6, 7]. On the other hand, “Healthcare Model” is related to care processes, trying to evaluate how well coordinated the care system is and fulfils patients’ needs. This is also linked with the underlying idea of functional integration and how it is evolving in the organisation.

As far as collaboration is concerned, there has also been a relevant improvement in almost all the items that comprise D'Amour Questionnaire, with no significant differences between the opinion of primary and secondary care professionals. Similarly, a very good performance at hospital utilisation (−7%) and Ambulatory Sensitive Care Conditions rates (−10%) has been achieved in the Bidasoa region. Moreover, patients report to receive coordinated care. Consequently, it can be stated that there is alignment in the evolution of the integrated care approach, collaboration and the outcomes achieved.

These results are consistent with available scientific evidence that shows how integrated care initiatives are able to bring positive results to the organisations where they are implemented [34]. However, it's not easy to make connections between causes and effects when it comes to establishing the factors that have contributed most to the improvements. Among the facilitators that have hastened the integration process, leadership is one of the most influential ones. A primary care-oriented management team (both the CEO and the Medical Director were Family Physicians), with a clear vision of the need to manage population health holistically, and with a strong emphasis on creating a culture of collaboration in which primary and secondary care shares a common language around patients’ needs is paramount. And this has been the key of the success of integration in Bidasoa. Furthermore, continuous communication with professionals at all levels and technology has also been a key factor in this process.

As evidence points out organisational integration by itself will not deliver benefits if clinicians do not change the way they work [4, 35]. The benefits of organisational integration become real only if all the players promote clinical and service integration. This is what Bidasoa Integrated Health Organisation has done through the development of a sense of belonging to the same organisation, communication, mutual knowledge and trust among health workers.

Even though Bidasoa Integrated Health Organisation seems to be improving health outcomes for its population, present results cannot guarantee its future results. Furthermore, more time should be given to properly assess the impact of the Integrated Health Organisation in Bidasoa. As we have seen, there has been slight progress in some of the indicators and this would require further analysis across a longer period of time. As it has been said of the challenges, such as cultural changes, do not happen from one day to another. It takes time to change to the way “things have always been done” and it would be normal to face some opposition among some of the stakeholders when implemented in bigger organisations. For instance, some health workers and managers from Primary Care see the integration process as a loss of power within the Organisation and perceive that most of the power has shifted to the Hospital. However, as we have seen in Figure 10, the percentage of total health spending is slowly moving from specialised to primary care. It is important to point out that Osakidetza/Basque Health Service is the biggest employer of the Basque Country with more than 30,000 people employed. This brings us to the point that it will be a challenge to maintain the structural changes over time in the medium term and long term. And this is why political support must continue over time and should not suffer from the changes in Government. Similarly, these results cannot be directly translated into the rest of IHOs in the Basque Country, since integrated care initiatives should be adapted locally [36].

To sum up, it is important to remember that the implementation of the “Strategy to Tackle the Challenge of Chronicity in the Basque Country” is the most important health reform being conducted since the 1980s. Not only has it served as the umbrella for other important changes (creation of IHOs, development of new nursing roles, patient activation programmes, etc.) but most importantly, it has set the baseline for a deeper integration in the health care delivery system.

## Conclusion

There is a significant amount of data that shows that Bidasoa Integrated Health Organisation has progressed in terms of delivering integrated care for chronic conditions with a positive impact on several Triple Aim outcomes. However, the methodology of this study does not allow us to demonstrate causality. These findings should be considered cautiously, but provide several interesting lessons for other organisations involved in care integration processes, like the multidimensional approach to integration (structural, functional and clinical) and the need to monitor carefully and assess the progression of the integration processes.

## Figures and Tables

**Table 1.**
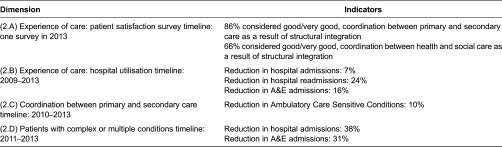
Patient care experience: an overview
